# A Complete Developmental Sequence of a *Drosophila* Neuronal Lineage as Revealed by Twin-Spot MARCM

**DOI:** 10.1371/journal.pbio.1000461

**Published:** 2010-08-24

**Authors:** Hung-Hsiang Yu, Chih-Fei Kao, Yisheng He, Peng Ding, Jui-Chun Kao, Tzumin Lee

**Affiliations:** 1 Howard Hughes Medical Institute, Janelia Farm Research Campus, Ashburn, Virginia, United States of America; 2 Department of Neurobiology, University of Massachusetts, Worcester, Massachusetts, United States of America; Baylor College of Medicine, United States of America

## Abstract

*Drosophila* brains contain numerous neurons that form complex circuits. These neurons are derived in stereotyped patterns from a fixed number of progenitors, called neuroblasts, and identifying individual neurons made by a neuroblast facilitates the reconstruction of neural circuits. An improved MARCM (mosaic analysis with a repressible cell marker) technique, called twin-spot MARCM, allows one to label the sister clones derived from a common progenitor simultaneously in different colors. It enables identification of every single neuron in an extended neuronal lineage based on the order of neuron birth. Here we report the first example, to our knowledge, of complete lineage analysis among neurons derived from a common neuroblast that relay olfactory information from the antennal lobe (AL) to higher brain centers. By identifying the sequentially derived neurons, we found that the neuroblast serially makes 40 types of AL projection neurons (PNs). During embryogenesis, one PN with multi-glomerular innervation and 18 uniglomerular PNs targeting 17 glomeruli of the adult AL are born. Many more PNs of 22 additional types, including four types of polyglomerular PNs, derive after the neuroblast resumes dividing in early larvae. Although different offspring are generated in a rather arbitrary sequence, the birth order strictly dictates the fate of each post-mitotic neuron, including the fate of programmed cell death. Notably, the embryonic progenitor has an altered temporal identity following each self-renewing asymmetric cell division. After larval hatching, the same progenitor produces multiple neurons for each cell type, but the number of neurons for each type is tightly regulated. These observations substantiate the origin-dependent specification of neuron types. Sequencing neuronal lineages will not only unravel how a complex brain develops but also permit systematic identification of neuron types for detailed structure and function analysis of the brain.

## Abstract 2

Labeling every neuron in a lineage in the fruit fly olfactory system reveals that every cell is born with a pre-determined cell fate that is invariant and dependent upon neuron birth order

## Author Summary

A brain consists of numerous, potentially individually unique neurons that derive from a limited number of progenitors. It has been shown in various model organisms that specific neurons arise in a lineage made by a repeatedly renewing progenitor at specific times of development. However, except in the worm *C. elegans*, the stereotype of neural development has never been examined in sufficient detail to account for every single neuron derived from a common progenitor. Here we applied a sophisticated genetic mosaic system to mark single neurons in the adult *Drosophila* brain and simultaneously reveal in which lineage a targeted neuron had arisen and when along the lineage it was made. We have identified each neuron in a lineage of olfactory projection neurons. There are a remarkable 40 types of neurons within this lineage born over two epochs. Strikingly, the birth order strictly dictates the fate of each post-mitotic neuron, including the fate of programmed cell death, such that every neuron type has a unique and invariant cell count. Sequencing an entire neuronal lineage provides definitive evidence for origin-dependent neuron type specification. It further permits a systematic characterization of neuron types for comprehensive circuitry mapping.

## Introduction

The brain consists of a great diversity of neurons derived from only a limited number of progenitors, called neuroblasts (NBs) [1]–[4]. Most NBs generate multiple neuron types [1],[5]–[7]. Notably, specific neurons are made by specific NBs at specific times of development, suggesting stereotyped patterns of neurogenesis [6]–[9]. High-resolution cell lineage analysis permits systematic identification of neuron types by resolving every single neuron in a neuronal lineage. Determination of neuron types based on their developmental origin will not only reveal the circuitry of the brain but also illustrate how a complex brain develops.

The clonal nature of brain development is particularly evident in organisms where neurons of the same clonal origin remain clustered in the mature brain [10]–[12]. The ability to recognize individual clones and follow their development has shed much light on the development and organization of the *Drosophila* central nervous system (CNS) [6],[7],[11],[12], in which NBs are individually identifiable [3],[4],[13]–[15]. They acquire region-specific cell fates and generate progeny whose projections are characteristic to each lineage [6],[7],[12],[16]. Neurons of a lineage derive sequentially: a given NB repeatedly undergoes asymmetric cell division to renew itself and produce a ganglion mother cell (GMC), which divides once to produce two mature neurons [17]. Such sister cells derived from a GMC may acquire distinct fates due to differential Notch signaling [18] and are further organized according to their hemilineage origin [19],[20]. Thus, most neuronal lineages consist of two hemilineages with distinct trajectories, and many grossly homogeneous lineages actually exist as lone hemilineages because their counterparts die during development through apoptosis [19]–[21].

A mature brain, comprised of a huge repertoire of diverse neurons, requires the production of multiple neuron types per hemilineage [6],[7],[16],[22]. The neurogenic diversity of holometabolous insects arises in two waves [15]: first, embryonically, most NBs produce primary neurons for wiring of larval circuitry [23], which may remodel during metamorphosis to contribute to the adult circuitry [24]–[26]; second, NBs generate adult-specific secondary neurons throughout larval development [6],[7],[12],[16]. A complete neuronal lineage can thus be divided into two discrete blocks, with multiple neuron types arising in a stereotyped pattern within each developmental epoch [7],[16],[26]. Birthdating of identifiable primary neurons in the embryonic ventral ganglion has revealed that unique neurons, at least for the first-few-born neurons, within a clone originate in an invariant sequence [27]. Diverse secondary neurons of the same hemilineage also derive sequentially in non-overlapping windows [7],[16]. Notably, the number of distinguishable cell types that derive in a given window may vary drastically in different lineages [6],[7],[16],[28]. Thus, distinct NBs produce multiple neuron types in different lineage-specific temporal patterns [6],[7],[16],[28], and sister hemilineages may even alter temporal identity at different tempos [29]. To identify all neuron types in such stereotyped lineages, one should identify every single neuron of each hemilineage based on the neuronal birth order.

**Figure 1:**
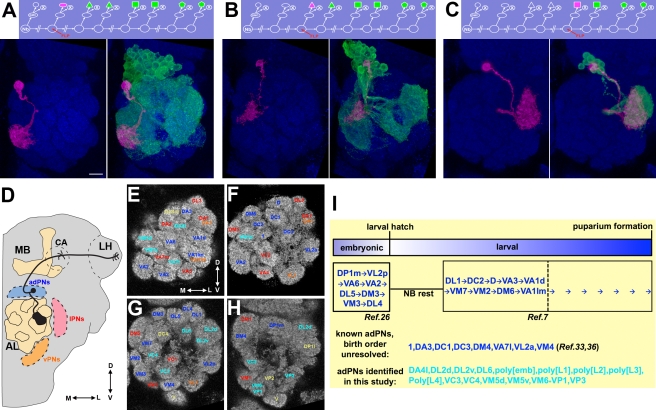
Examples of paired sister clones, the AL glomerular architecture, and a summary of adPNs. (A–C) GMC clones (magenta) pairs with NB clones (green) of different sizes depending on when mitotic recombination occurred in a protracted lineage. Judging from the size of the accompanying NB clone, one can determine when the GMC of a particular neuron was born in the lineage. One can therefore deduce in the adPN lineage: the VM3-targeting neuron (magenta in [A]) born around the beginning, the 1-targeting neuron (magenta in [B]) derived in the middle, and the DL2v-targeting neuron (magenta in [C]) made near the end. (D) A schematic illustration of an uniglomerular adPN (black) that connects one of the 50 or so AL glomeruli with the calyx (CA) of the mushroom body (MB) and the lateral horn (LH). The relative position of three populations of PNs, the adPNs (blue), lPNs (red), and vPNs (orange), is also shown. (E–H) All identifiable glomeruli in the AL are shown in four anterior-to-posterior focal sections. The glomerular targets of previously identified uniglomerular adPNs, lPNs, and vPNs are labeled in blue, red, and orange, respectively. The glomerular targets of the uniglomerular adPNs identified in this study are shown in cyan. And the glomeruli with yellow labels have not yet found their corresponding uniglomerular PNs. (I) adPNs identified previously and in this study are summarized. The adPNs with known birth order are further arranged with respect to the lineage development. Fly brains were counterstained with nc82 mAb (blue) in this and all other figures, which permits determination of glomerular identity in the AL. The scale bar in this and all other figures equals 10 µm.

An improved MARCM (Mosaic Analysis with a Repressible Cell Marker) technique, called twin-spot MARCM, permits high-resolution cell lineage analysis [28]. Following mitotic recombination, twin-spot MARCM labels sister clones in distinct colors in otherwise unstained tissue. In a typical neuronal lineage, a twin-spot MARCM clone reveals two populations of cells: one or two neurons derived from the GMC paired with all of the later-born neurons in the lineage, which is labeled as the NB clone (e.g., Figure 1A). Counting the cell number of an NB clone reveals the temporal position of its paired neuron(s) along the lineage (e.g., Figure 1A–C). Also, analysis of NB clones of all sizes in a stereotyped lineage should reveal the order in which the post-mitotic neurons of the lineage have been derived. Thus, a complete description of neuron composition of the *Drosophila* brain can be reached by identifying every single neuron in all lineages. Such analysis will also uncover all neuronal trajectories and elucidate the number of the same type of neurons that have indistinguishable morphology.

Stereotyped lineages underlie the development of the *Drosophila* antennal lobe (AL), where a topographic map of olfaction is established between the peripheral olfactory receptor neurons (ORNs) and the AL projection neurons (PNs) (Figure 1D) [7],[30]–[33]. There are about 50 glomeruli in the adult AL (Figure 1E–H) [34]. All ORNs expressing the same odorant receptor project to the same glomerulus, where they synapse with PNs; ORNs expressing different odorant receptors project to distinct glomeruli [32],[35]–[39]. Many PNs, like ORNs, target only one AL glomerulus [22],[33]. PNs send axons to higher brain centers, including the mushroom body (MB) and the lateral horn (LH) (Figure 1D) [22],[33]. Distinct PNs further acquire different characteristic patterns of axon projections [22],[33]. Following the trajectories of PNs that connect with distinct ORNs has started to unravel how different olfactory inputs might be processed differentially to govern diverse organismal behaviors [35]. However, in contrast with a near-complete description of ORNs [38],[39], the uniglomerular PNs of several AL glomeruli, if they exist, remain to be identified [22],[26],[33]. In addition, there possibly exist diverse types of polyglomerular PNs that may relay specific patterns of olfactory inputs to higher brain centers [22],[29],[40]. Therefore, the olfactory topographic map of the adult AL will not be complete until all PN types have been identified and counted.

Here we determined every single neuron in an AL PN lineage through analysis of numerous twin-spot MARCM clones. We uncovered 15 additional PN types, including five polyglomerular types of PNs in the otherwise pure uniglomerular lineage; these distinct PNs are born in an invariant sequence. Notably, the NB alters temporal identity following each embryonic division and yields 18 types of PNs during its brief production of primary neurons. In contrast, only 22 morphologically distinguishable types of PNs derive from the many more secondary neurons generated by the same NB. Furthermore, these larval-born multi-neuronal cell types show specific cell counts, suggesting the tightly regulated fate of individual neurons chronologically as well as spatially and supporting the functional significance of these neurons. This is the first study, to our knowledge, to completely describe the neuron composition of a neural lineage, and this also underscores the importance of deciphering individual neurons in all lineages to elucidate the brain development and function.

## Results

### Strategies for Sequencing the AL PN Lineage That Can Be Selectively Targeted by *GAL4-GH146* and *acj6-GAL4*

Three AL PN lineages have been partially characterized [7],[22],[26],[29],[33],[41]. Among them, the anterodorsal PN (adPN) lineage is best studied [7],[19],[22],[26],[33]. It exists as a lone hemilineage and can be fully covered with *acj6-GAL4* [19],[29],[42]. In addition, many adPNs are positive for *GAL4-GH146*. Twenty-five types of uniglomerular PNs have been identified through single-cell analysis of GH146-positive adPNs [7],[22],[33],[36]. Distinct adPNs derive in an invariant sequence [7],[26]. However, their birth order has not been completely resolved. In addition, the number of neurons comprising the lineage is unknown. Finally, GH146-negative adPNs have only been treated as a population [29], and while some glomeruli innervated by these PNs have been identified, the projection patterns of individual GH146-negative adPNs remain undetermined.

To resolve the entire lineage based on neuronal birth order, we first determined when most of the elusive GH146-negative adPNs were born through analysis of adPN NB clones induced at different times of development. Dual-expression-control MARCM allowed us to label GH146-positive adPNs of the clones with *LexA::GAD-GH146* while marking the entire clones using *acj6-GAL4* [29],[43]. We obtained similar numbers of GH146-negative cells among the dual-expression-control MARCM clones, even when clones were induced at the mid-3rd instar larval stage, labeling only the last VA1lm-targeting GH146-positive adPN and subsequently born neurons in the adPN NB clone (around 32 cells; *n* = 5; Figure S1). In these clones, six glomeruli were prominently marked by the GH146-negative neurons (Figure S1B). These observations suggest that most, if not all, GH146-negative adPNs derive after birth of their GH146-positive siblings and that they innervate a distinct set of glomeruli from the GH146-positive adPNs.

**Figure 2:**
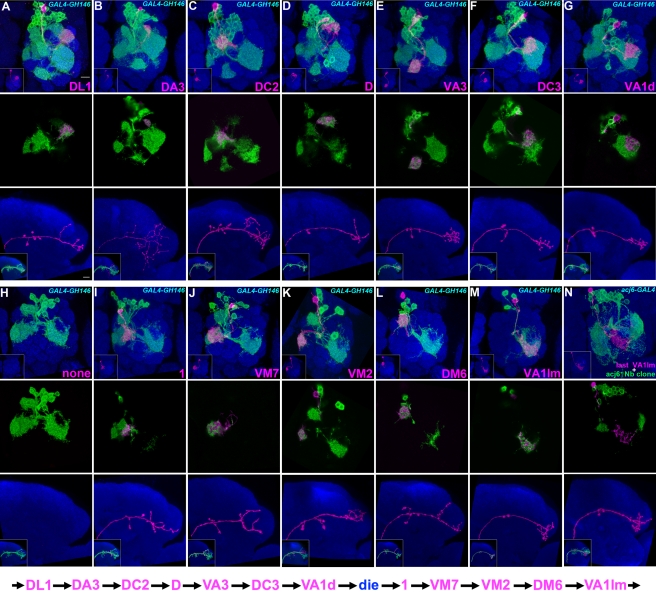
Twelve types of early-larval-derived GH146-positive uniglomerular adPNs. Twin-spot MARCM clones of adPNs labeled with *GAL4-GH146* (A-M) or *acj6-GAL4* (N). Top panels: composite confocal images of sister clones in the AL; middle panels: single focal sections of the AL covering the glomerular targets of GMC progeny (magenta); bottom panels: axon projections of GMC progeny (magenta); islets in bottom panels: axon projections of both GMC progeny (magenta) and its paired NB clone (green). Note each adPN type (magenta) consistently pairs with adPN NB clones (green) of specific compositions. Analysis of NB clones revealed the 12 types of GH146-positive adPNs are made in an invariant sequence from (A) to (M). And all the lone, unpaired NB clones (H), whose preceding GMC progeny probably die prematurely, were induced in the interval between VA1d and 1 adPNs. The sequence of early-larval adPN neurogenesis is summarized in the bottom. In addition, there are multiple neurons per type, as evidenced in middle panels that the glomerular target of GMC progeny can be co-labeled by its accompanying NB clone. For the lineage after VA1lm PNs, one can visualize GH146-negative adPNs with *acj6-GAL4* as revealed in (N) where the last VA1lm adPN pairs with a 32-neuron-containing NB clone.

**Figure 3 pbio-1000461-g003:**
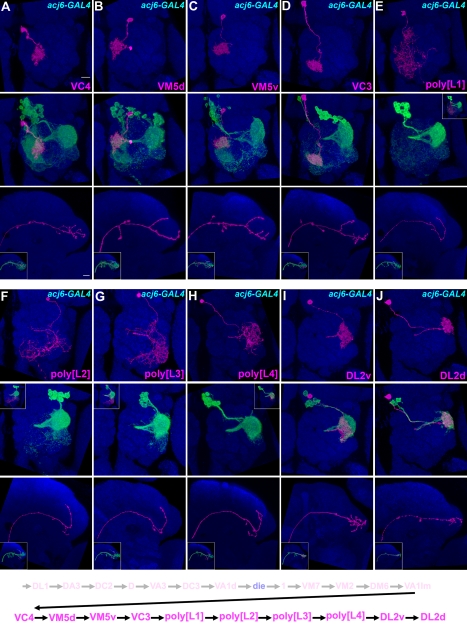
Ten types of late-larval-derived Acj6-positive adPNs. Late-larval-derived twin-spot clones labeled with *acj6-GAL4*. Top and middle panels: composite confocal images of the AL showing single adPNs only or both single adPNs (magenta) and their paired NB clones (green); bottom panels: the axon projections of single adPNs and in islets the projections of single adPNs (magenta) and their accompanying NB clones (green). Analysis of NB clones paired with distinct adPNs revealed 10 additional adPN types are made in an invariant sequence as summarized in the bottom. Note presence of four types of polyglomerular PNs that exhibit different patterns of AL elaboration while acquiring analogous axon projections in the LH (E–H).

**Figure 4:**
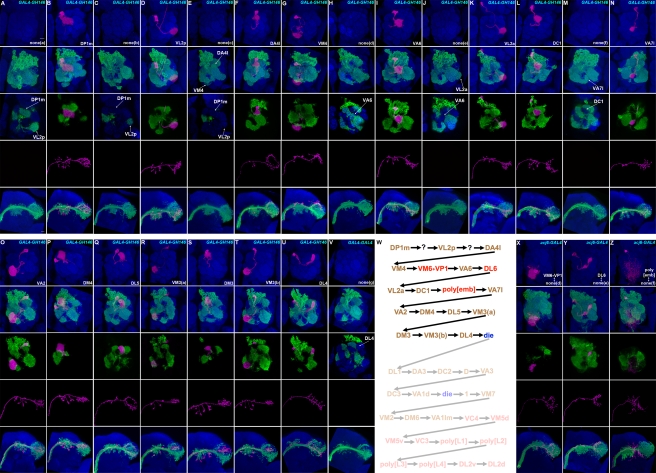
Eighteen types of embryonic-born adPNs. Embryonic-derived twin-spot clones labeled with *GAL4-GH146* (A–V) or *acj6-GAL4* (X–Z). Top two panels: composite confocal images of the AL showing single adPNs (magenta) and the paired NB clones (green); middle panels: single focal sections of the AL covering the glomerular targets of single adPNs; bottom two panels: axon projections of single adPNs (magenta) and the accompanying NB clones (green). 22 types of GH146-labeled NB clones can be distinguished and are shown in the order of decreasing complexity from (A) to (V). 15 of them pair with distinct adPNs while seven of them exist alone (no magenta labeling). The unpaired NB clones in (H), (J), and (M), when labeled with acj6-GAL4, were paired with novel GH146-negative adPNs, including one additional type of polyglomerular PN (X–Z). The sequence of embryonic-derived adPNs is shown in (W). Note presence of one neuron per type (except VM3-targeting PNs in [R] and [T]) in the lineage of primary neurons, as evidenced by the glomerular targets of single adPNs (magenta) not innervated by their accompanying NB clones (green).

We supposed that use of *GAL4-GH146* alone should allow us to identify the majority of adPNs born prior to the mid-larval stage. We therefore reserved *acj6-GAL4*, which has much more non-PN expression, for the elucidation of the late larval development of the adPN lineage and filling any gap present in the sequence of GH146-positive adPNs. As described below, we first resolved the GH146 part of the larval adPN lineage (Figure 2). We then determined the later-derived GH146-negative adPNs using *acj6-GAL4* (Figure 3). Finally, we identified the embryonic-born adPNs with *GAL4-GH146*, followed by *acj6-GAL4* (Figure 4).

### The Early Larval adPN NB Serially Generates 12 Uniglomerular PN Types and, in a Specific Interval, Some Programmed-to-Die Cells

To identify the larval-derived GH146-positive adPNs as well as determine their birth order, we obtained numerous adPN NB clones paired with distinct PNs following induction of mitotic recombination at different times of larval development. The NB clones may pair with one of the following 12 types of uniglomerular PNs: DL1, DA3, DC2, D, VA3, DC3, VA1d, 1, VM7, VM2, DM6, and VA1lm (in the order of disappearance from the NB clones of decreasing sizes; see below; Figure 2A–M). Four previously identified GH146-positive adPN types, including DC1, DM4, VL2a, and VM4, were never detected among the paired single-cell clones [33]. These indicate production of specific adPN types during early larval development.

We then determined if specific adPNs are derived from GMCs born at specific windows of the lineage. Because distinct PNs target different AL glomeruli, the offspring composition of a multi-cellular adPN NB clone can be inferred based on the labeled AL glomeruli. Thus, we determined which neurons remained to be derived after birth of a particular PN by analyzing the offspring composition of its paired NB clone. Using this method, we found in every DL1-paired NB clone the presence of all the remaining 11 types of uniglomerular PNs, arguing that the birth of DL1 adPNs precedes the others' (the top panel of Figure 2A; *n*>50). The DL1 glomerulus was often targeted by the DL1 single neuron as well as its paired NB clone, suggesting the presence of multiple DL1 adPNs (the middle panel of Figure 2A). Moreover, all the DL1 PNs are born before the NB transits to make other PN types, since we rarely detected the DL1 glomerulus in the NB clones paired with other larval-derived adPN types (<1%, n>100; unpublished data; such rare events possibly resulted from contamination with GMC-derived single-cell clones). Annotation of the AL glomerular targets of the adPN NB clones paired with each of the remaining 11 types of uniglomerular PNs subsequently revealed the order in which the 12 types of GH146-positive adPNs derive during early larval development (unpublished data; the analysis was done similarly as shown in Table S1). Briefly, the DL1, DA3, DC2, D, VA3, DC3, VA1d, 1, VM7, VM2, and DM6 glomerulus correspondingly disappeared from the adPN NB clones paired with the DA3, DC2, D, VA3, DC3, VA1d, 1, VM7, VM2, DM6, and VA1lm PN (Figure 2B–G and 2I–M; *n*>4 for each). This indicates an orderly derivation of 12 types of adPNs from a common progenitor.

In addition, we noticed the presence of unpaired NB clones that consistently carry the last five types of GH146-positive adPNs (Figure 2H; *n* = 5). This indicates the absence of GMC progeny in the interval between the generation of adPNs innervating VA1d and 1. The same gap was also evident when we marked a similar pool of adPN twin-spot clones using the pan-adPN driver, *acj6-GAL4* (unpublished data). Since no acj6-positive, GH146-negative adPN was born in the same window, the post-mitotic neurons made between the VA1d and 1 adPNs are probably programmed to die. Taken together, the early larval adPN NB serially makes 12 uniglomerular PN types and, in a specific interval, some prematurely lost cells.

### Six Types of Uniglomerular PNs Plus Four Types of Polyglomerular PNs Subsequently Derive After the GH146-Positive Offspring

We next determined the subsequently derived GH146-negative adPNs and their birth order through analysis of twin-spot clones labeled with *acj6-GAL4*. The *acj6-GAL4*-labeled NB clones that paired with the last sibling of VA1lm-targeting adPNs, the end of GH146-positive series, contain about 32 post-mitotic neurons (Figure 2N; *n* = 3). Similar cell numbers were obtained from counting the GH146-negative cells among the dual-expression-control MARCM clones (Figure S1; *n* = 5). A distinct set of six AL glomeruli, including VC4, VM5d, VM5v, VC3, DL2v, and DL2d, were prominently labeled in the largest NB clones exclusively negative for *GAL4-GH146* (Figure 2N and Figure S1B). This suggests probably six novel types of uniglomerular PNs are made in the remaining adPN lineage.

Analysis of single-cell clones of acj6-positive, GH146-negative adPNs generated in late larvae revealed the six types of uniglomerular PNs (Figure 3A–D and 3I–J; *n*>4 for each). Interestingly, we also identified PNs that spread neurites across multiple glomeruli (Figure 3E–H; *n*>4 for each). Four distinct polyglomerular patterns, tentatively referred to as poly[L1], poly[L2], poly[L3], and poly[L4], could be detected (Figure 3E–H). They innervate partially overlapping subsets of eight specific AL glomeruli. The four patterns from poly[L1] to poly[L4], respectively, cover: (1) parts of DC4, VC3, and VM4 (Figure 3E); (2) VM4, VL2p, and regions posterior to medial ventral AL (Figure 3F); (3) VL2p, VL2a, and DL1l (Figure 3G); and (4) parts of VL2a, DL1l, DL2v, and DL2d (Figure 3H). Notably, such polyglomerular adPNs exhibit similar trajectories in the LH despite differences in the AL elaboration (bottom panels of Figure 3E–H). This is in sharp contrast with their uniglomerular siblings whose LH trajectories differ among PNs targeting distinct glomeruli (e.g., Figures S4–S5) [7],. This might lead one to wonder if different polyglomerular patterns may result from developmental plasticity rather than being precisely prespecified. Nonetheless, closer inspection permitted identification of these four types of polyglomerular PNs even in NB clones (the middle panels in Figure 3E–H). They disappeared again in an invariant sequence from the adPN NB clones of decreasing sizes (see below), demonstrating the presence of four polyglomerular PN types in the otherwise uniglomerular PN lineage.

Analysis of adPN NB clones paired with distinct PNs, including the six additional types of uniglomerular PNs and the four types of polyglomerular PNs, further revealed that these 10 types of GH146-negative PNs are consistently derived in the following specific order: VC4, VM5d, VM5v, VC3, poly[L1], poly[L2], poly[L3], poly[L4], DL2v, and DL2d. Notably, the four types of polyglomerular PNs are generated contiguously in the interval between the production of VC3 and DL2v uniglomerular PNs (Figure 3D–I). In other words, no gap exists in the GH146-negative sublineage; unpaired adPN NB clones are observed only in the earlier window when the adPN NB transits from making the VA1d PNs to yielding the 1 PN (Figure 2G–I). Taken together, through larval development, the adPN NB makes 22 types of AL PNs, including 18 uniglomerular PN types and four polyglomerular PN types.

### 18 Uniglomerular PNs, Targeting 17 Additional AL Glomeruli, Plus One Polyglomerular PN Constitute the Embryonic adPN Lineage

Following the determination of secondary neurons, we examined the primary neurons generated by the adPN NB. We first determined the GH146-positive adPNs born in embryos. Clones were induced during embryogenesis and labeled by twin-spot MARCM with *GAL4-GH146*. We uncovered 14 additional types of uniglomerular PNs through analysis of embryonic-derived, single-cell clones of adPNs. They innervate one of 14 adult AL glomeruli, including DP1m, VL2p, DA4l, VM4, VA6, VL2a, DC1, VA7l, VA2, DM4, DL5, DM3, VM3, and DL4 (in the order of disappearance from the NB clones of decreasing sizes; see below; Figure 4A–V and Figure S2; Table S1). We further obtained the adPN NB clones that pair with each of the 14 types of primary neurons (Figure 4B, 4D, 4F–G, 4I, 4K–L, and 4N–U). Intriguingly, the embryonic-derived adPN NB clones, except those paired with VM3-targeting adPNs (Figure 4R and 4T), never innervated the same glomerulus labeled by the GMC side of twin spots. This indicates presence of only one adPN for the majority of primary-neuron-targeted glomeruli. This stands in great contrast to the larval-derived adPN types that consistently exist in multiple-cell groups, as evidenced by their glomerular targets often being co-labeled by both NB and GMC sides of twin spots (Figures 2–3).

We then analyzed the detailed glomerular innervation patterns of the NB clones. Notably, the 14 glomeruli targeted by the primary neurons disappeared in a stereotyped order as the clone size decreased. This reveals derivation of distinct primary neurons in an invariant sequence as well. It further shows production of VM3 adPNs in two windows separated by the birth of the DM3 adPN (Figure 4R and 4T). There should be only one VM3 adPN born after the DM3 adPN, since the VM3 glomerulus was never co-labeled by both sides of twin spots after birth of the DM3 adPN (Figure 4S–T). Indeed, a lone VM3 adPN consistently innervates the VM3 glomerulus in coarse patches (the middle panel of Figure 4T). Notably, the VM3 glomerulus was fully tiled upon co-labeling by an earlier-derived VM3 adPN within the accompanying NB clone (the middle panel of Figure 4R). This suggests presence of only one VM3 adPN born before the DM3 adPN as well. Besides innervating the same glomerulus in complementary patches, they exhibit similar patterns of axon arborization in the LH (Figure 4R and 4T). This argues for the presence of two indistinguishable VM3 adPNs, despite their derivation in distinct windows. In sum, the embryonic adPN NB makes 15 GH146-positive uniglomerular PNs in the following order: DP1m, VL2p, DA4l, VM4, VA6, VL2a, DC1, VA7l, VA2, DM4, DL5, VM3(a), DM3, VM3(b), and DL4.

In addition, we obtained seven classes of unpaired NB clones that show specific patterns of glomerular innervation, suggesting the presence of some GH146-negative primary neurons and/or premature loss of certain primary neurons (Figure 4A, 4C, 4E, 4H, 4J, 4M, and 4V). Analysis of twin-spot adPN clones marked with *acj6-GAL4* subsequently allowed us to identify two additional uniglomerular PNs and one polyglomerular PN that fill three of the seven gaps in the GH146-positive primary neuron sequence (Figure 4X–Z; Table S2). The VM6+VP1 PN lies in the gap between the VM4 adPN and the VA6 adPN (Figure 4X); the DL6 PN resides between VA6 and VL2a (Figure 4Y); and the polyglomerular PN derives in the interval between DC1 and VA7l (Figure 4Z).

Despite use of the pan-adPN driver *acj6-GAL4*, we obtained two classes of unpaired NB clones (Figure 4A, 4V and unpublished data; Table S2). These occurred following mitotic recombination at the beginning of the lineage and at the end of primary neuron production, respectively. The lone largest clones were probably hit during the birth of the adPN NB, and their sister clones could reside outside the CNS (Figure 4A). In contrast, the lone NB clones that exclusively consist of the entire secondary lineage should pair with the last-born primary neuron, absence of which indicates another neuronal loss in the protracted adPN hemilineage (Figure 4V).

The remaining two gaps in the sequence of GH146-positive adPNs were unresolved following analysis of all the acj6-labeled NB clones induced during embryogenesis (Table S2). These should be occupied by the 2nd and 4th sibling, respectively, but we did not obtain acj6-labeled NB clones hit during the birth of their precursors. Therefore, the fate of these post-mitotic neurons remains to be determined. Nonetheless, detailed analysis of several acj6-labeled, full-sized NB clones (Figure S3A–S3D) allowed us to detect VP3 as another glomerular target of adPNs (Figure S3D). We could also obtain single-cell clones of VP3-targeting PNs when clones were labeled with *acj6-GAL4* (Figure S3E). Further, the VP3 adPN was absent from the NB clones paired with the fifth or any later-derived adPN (unpublished data). Since no additional uniglomerular adPN could be found, the VP3 adPN and possibly another premature-lost neuron might account for the final two gaps in the otherwise completely resolved adPN sequence.

Taken together, the embryonic adPN NB invariantly makes 19 viable PNs, including 18 uniglomerular PNs of 17 types and one polyglomerular PN, from a possible set of 21 asymmetric cell divisions. There is minimal cellular redundancy with one neuron per glomerular target except the VM3 glomerulus. This is in great contrast with the secondary neurons of the same lineage, which outnumber the primary neurons by 3- to 4-fold but only add a comparable number of PN types (22 versus 18) to the mature adPN lineage.

### Multi-Cellular PN Types Show Stereotyped Cell Counts

**Figure 5:**
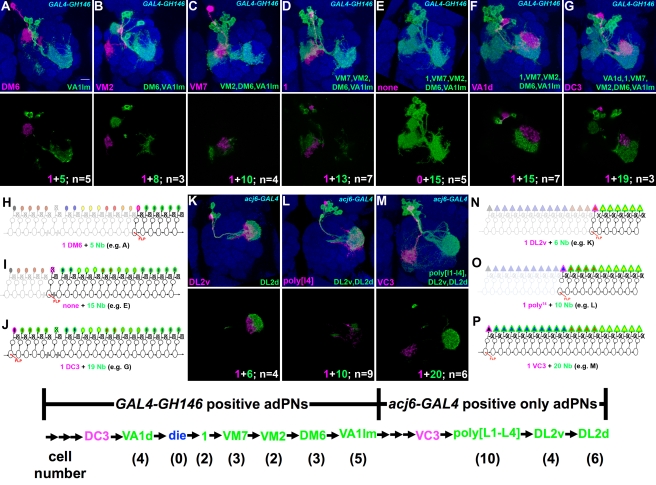
Distinct cell counts in different adPN types. Twin-spot clones labeled with *GAL4-GH146* (A–G) or *acj6-GAL4* (K–M). Upper panels: composite confocal images of sister clones in the AL; lower panels: single focal sections showing no innervation of the magenta glomeruli by the green NB clones, indicating clones derived during birth of the last sibling of the preceding adPN type. The clones shown in (A) and illustrated in (H) reveal the adPN NB makes five VA1lm-targeting PNs following derivation of the last DM6-targeting PN. Illustrations of lineage development for additional twin-spot clones are shown in (I), (J), and (N) to (P). Invariant cell counts were obtained for the majority of NB clones paired with the last sibling of the preceding adPN type (see Tables S3 and S4). These support production of a fixed number of neurons for each multi-cellular adPN type, as summarized in the bottom.

Because the AL glomeruli innervated by primary versus secondary neurons operate through solo or multiple PNs, we wondered if fixed cell counts exist for the multi-cellular adPN types composed of secondary neurons. To answer this question, one needs to count the cells of NB clones. We first analyzed GH146-labeled NB clones and started with the ones homogeneously consisting of VA1lm PNs (the last-derived GH146-positive adPN type; Figure 5A; Table S3). All the NB clones that paired with the last DM6 sibling (the preceding adPN type) and thus carrying an entire set of VA1lm adPNs had five cell bodies. This indicates that there are always five VA1lm PNs made by the adPN NB. And the NB clones paired with the last VM2 sibling (the adPN type preceding DM6) possessed eight neurons that include five VA1lm and three DM6 uniglomerular PNs (Figure 5B; Table S3). In this way, we worked backward to determine the cell numbers for late- to early-derived adPN types. Invariant cell counts were obtained for the majority of NB clones paired with the last sibling of six contiguous PN types, which allowed us to deduce that the adPN NB consistently makes 5 VA1lm, 3 DM6, 2 VM2, 3 VM7, 2 1, and 4 VA1d PNs (Figure 5A–G; Table S3).

Analysis of acj6-labeled NB clones using the same strategy revealed invariant cell fates for the last-born 21 adPNs as well. After making the last VC3 uniglomerular PN, the NB serially yielded 10 polyglomerular, 4 DL2v, and 6 DL2d PNs as the lineage ends by pupation (Figure 5K–M; Table S4). The polyglomerular patterns of innervation could only be unambiguously discerned in single-cell clones, preventing further cell counts for specific polyglomerular types. In conclusion, the adPN NB not only makes specific types of AL PNs but also generates a fixed number of neurons for each PN type.

## Discussion

The AL receives odorant inputs from ORNs that reside in two peripheral appendixes, the antennae and maxillary pulps. Identification and characterization of various types of PNs and local interneurons (LNs) in the AL has enhanced our understanding of how the olfactory information is relayed and integrated in the *Drosophila* brain [7],[22],[26],[29],[33],[41],[44]. Given the stereotypy of neural development, comprehensive cell lineage analysis, which is made possible by twin-spot MARCM [28], should allow one to identify all AL PNs and LNs systematically. Here we have determined every single neuron within one of five AL lineages (see below for the description of these five AL lineages). Forty types of AL PNs derive in an invariant sequence in the adPN lineage (Figures 2–4) and diverse PN types further show distinct, specific cell counts (Figure 5). These argue that the fate of individual neurons in the adPN lineage is tightly regulated during development and possibly hint at the functional significance of these neurons in the olfactory circuit.

Clonal analysis using ubiquitous drivers has allowed us to uncover five neuronal lineages that generate neurons predominantly innervating the AL (Yu and Lee, unpublished observation). These include the previously reported adPN, lALN, and vPN lineages [7],[41], in addition to the vLN and lvPN lineages (Yu and Lee, unpublished observation). The vLN lineage generates LNs with cell bodies located ventral to the AL, while the adPN, lvPN, and vPN lineages produce PNs with cell bodies residing anterodorsal, lateroventral, and ventral to the AL, respectively. By contrast, the lALN lineage makes both LNs and PNs with cell bodies clustered lateral to the AL. Single-cell analysis has so far revealed that, excepting the lALN lineage, the remaining four lineages are homogeneously composed of AL neurons. By contrast, characterization of about 85 additional central brain lineages revealed only one lineage that shows sparse AL innervation (Yu and Lee, unpublished observation). These observations suggest that a specific set of neuronal lineages are selectively devoted to the AL development. Comprehensive lineage analysis to identify every single neuron within the five AL lineages should facilitate the reconstruction of neural circuitry in the primary olfactory center.

The AL relays olfactory information from ORNs to higher brain centers primarily through PNs that connect 52 AL glomeruli to the MB calyx and the LH. Assuming the presence of uniglomerular PNs for all AL glomeruli, 52 types of uniglomerular PNs are anticipated. Complete sequencing of the adPN lineage reveals 35 of them, and analysis of GH146-positive PNs in the lALN lineage has shown 12 additional types of uniglomerular PNs [33]. Notably, we identified a GH146-negative adPN innervating a previously unreported glomerulus (DL6; Figure 1Y) while finding another GH146-negative adPN occupying two vaguely separate glomeruli (VM6+VP1; Figure 1X) whose input(s) remains unknown [38]. We have thus modified the list of 52 AL glomeruli by adding DL6 and combining VM6 with VP1 (Figure 4X–Y), leaving the total number of AL glomeruli unchanged. Note that the “DL6” uniglomerular PN first mentioned in an earlier MARCM study of GH146-positive adPNs innervates DL4 rather than a previously unreported glomerulus [26]. Our study leaves five AL glomeruli, DA4m, V, DC4, VP2, and VL1, whose corresponding MB/LH-targeting uniglomerular PNs remain to be uncovered. The missing types of uniglomerular PNs should exist: with the exception of VP2, the olfactory inputs for these glomeruli are known and comparable to those innervating other AL glomeruli [38],[39]. Identifying the entire set of uniglomerular PNs and further determining their connectivity in the LH will help elucidate how odors govern organismal behavior.

Besides uncovering 10 previously unreported uniglomerular PN types, sequencing the entire adPN lineage has unexpectedly led to the discovery of a new class of polyglomerular PNs. Only a few polyglomerular PN types had been identified previously [22],[29]. They differ from uniglomerular PNs not only in the pattern of glomerular innervation but also in the trajectory of their axons. In contrast with the above 47 types of uniglomerular PNs that extend axons through the iACT and consistently target both the MB and LH (Figures 2–4) [22],[33], the axons of earlier identified polyglomerular PNs take distinct paths and innervate diverse targets, including the LH but not the MB [22],[29]. Interestingly, the five types of polyglomerular adPNs that we identify here navigate through the iACT like their uniglomerular siblings and potentially innervate both the MB and LH. However, they rarely made branches or synaptic boutons within the MB calyx (Table S5). Their axons appear to simply pass through the MB calyx and exclusively target the LH (e.g., Figures 3E–H and 4Z). Given the distinction, polyglomerular PNs probably serve different functions from the much better known uniglomerular PNs. Notably, the four contiguously born polyglomerular adPN types exhibit dendritic tiling within the AL and further acquire an indistinguishable axon trajectory in the LH (Figure 3E–H). Interestingly, their glomerular targets selectively receive inputs from the coeloconic type of sensilla [38]. Although the ORNs of coeloconics have just begun to be resolved [39], we propose that these polyglomerular PNs jointly modulate some innate behavior(s) in response to specific olfactory inputs.

Sequencing an entire lineage further revealed that the cell numbers of specific neuron types are tightly regulated (Figures 4–5). Embryonic-born adPN types have only a single neuron, with the exception of two neurons innervating VM3 (Figure 4). In contrast, all the adPN types derived after larval hatching are made up of multiple neurons (Figures 2–3). No difference in the size of glomeruli or quality of the olfactory input could be discerned between the single- and multi-PN glomeruli, and glomeruli instead show considerable variety: a lone uniglomerular PN can densely fill a large glomerulus (e.g., DP1m and VA2 in Figure 4B and 4O), while some small glomeruli are tiled by multiple PNs (e.g., DA3, VM2, and 1 in Figure 2B, 2I, and 2K). However, among the multi-PN glomeruli, the glomerular size does roughly correlate with its number of uniglomerular PNs. For example, the VA1lm and VA1d glomeruli are respectively innervated by five and four adPNs and are much more prominent than the VM2 and 1 glomeruli, both tiled by only two adPNs (Figure 5). Thus, the role of added PNs innervating a given glomerulus is not simply to allow for dendritic tiling and may play a role in mechanisms of olfactory coding.

We have further demonstrated the longstanding observation that adPNs possess distinct stereotyped axonal projection patterns within the LH (Figures S4–S5) [22],[26],[33]. However, we have also shown that PNs with the same glomerular target exhibit nearly indistinguishable patterns of axon arborization. When two adPNs were differentially marked as single-cell clones by twin-spot MARCM, we frequently observe among neurons targeting the same glomerulus co-migration of neurites and co-localization of bouton-like structures in the LH (Figure S4A–S4F compared with S4G–S4H). The same phenomena apply to the two separately derived VM3-targeting adPNs, arguing that contiguous birth is not a prerequisite for such coordinated projections (Figure 4R, 4T; Figure S5G and S5I). These data support the presence of multiple anatomically equivalent neurons per larval-derived adPN type.

Finally, neuronal birth order strictly dictates the fate of each post-mitotic neuron. This mechanism of predetermined cell fates nicely explains the observed stereotyped development of the adPN lineage, including uniglomerular PNs targeting one of the 52 AL glomeruli (Figures 2–4), premature cell loss (Figure 2H), and polyglomerular PNs that tile specific glomeruli in an invariant pattern while sending axons to the same probable target(s) (Figure 3E–H). Development clearly guides how neural circuitry is built, which, in turn, shapes development through evolution. Like other primary neurons, most adPNs born during embryogenesis are individually unique and have elaborated exuberantly enough to serve the functions demanding multiple secondary neurons (Figure 4). In contrast, secondary neurons are made in blocks during larval development with multiple cells acquiring the same fate within each block (Figures 2–3). Notably, post-embryonic NBs alter their temporal identity in lineage-specific patterns. Such developmental programs not only control neuron types but also confer cell counts to each neuron type and constrain how the circuitry is built. To elucidate why AL PN numbers, including those of polyglomerular types, are tightly controlled will require determination of the physiological consequences of perturbing cell numbers. To comprehensively determine neuron types based on developmental origin should reveal the organization of the entire brain, and to investigate how stereotyped lineages are made and further gain the ability to engineer their development will advance our understanding of the ultimate mechanism of function of the brain.

## Materials and Methods

### Fly Strains

The fly strains used in this study were: (1) *Acj6-GAL4* [42]; (2) *FRT^40A^,UAS-Cd2::rfp,UAS-gfp-Mir/CyO,Y* [28]; (3) *FRT^40A^, UAS-Cd8::gfp,UAS-Cd2-Mir/CyO,Y* [28]; (4) *FRT^40A^,UAS-Cd8::gfp,UAS-Cd2-Mir,GAL4-GH146/CyO,Y*; (5) *FRT^40A^,UAS-Cd8::gfp,lexAop-Cd2-gfp,LexA::GAD-GH146/CyO,Y*; (6) *hs-FLP[1]* [45]; (7) *hs-FLP[122]* [26]; and (8) *FRT^40A^,tubP-GAL80/CyO,Y* [46].

### Clonal Analysis with Dual-Expression-Control MARCM and Twin-Spot MARCM

The generation, dissection, immunostaining, and mounting of mosaic clones of adult brains have been described [46]. For dual-expression-control MARCM experiments, mosaic clones were induced using *hs-FLP* [*1*] at early larval and mid-3rd instar larval stages by heat-shock for 20 and 35 min, respectively. Determination of the birth order of PNs in Figures 2–4 is based on the cell number and neuronal composition of their paired NB clones, so samples were only roughly synchronized for heat shock at various later time points in twin-spot MARCM experiments. In short, mosaic clones of embryonic-born PNs were generated with *hs-FLP[122]* by collecting embryos for 18 h in vials and following with heat-shock for 10 min. Mosaic clones of larval-derived PNs were induced with either *hs-FLP* [*1*] or *hs-FLP[122]* by collecting embryos for 12 h in vials and heat-shock for 20–40 min at different developmental stages (every half day from 0.5 d after embryo collection to puparium formation). For example, DL1 paired with its NB clone in Figure 2A can be generated at 0.5–2 d after embryo collection. DA3 paired with its NB clone in Figure 2B can be generated at 2 d after embryo collection. VA1lm paired with its NB clone in Figure 2M can be generated at 4 d after embryo collection. DC2, D, VA3, DC3, VA1d, 1, VM7, VM2, and DM6 paired with their NB clones in Figure 2C–O can be generated between 2.5 d to 4 d after embryo collection. VC4, VM5d, VM5v, VC3, poly[L1], poly[L2], poly[L3], poly[L4], DL2v, and DL2d paired with their NB clones in Figure 3 can be generated between 4 d to puparium formation after embryo collection. To simplify the analysis of twin-spot MARCM clones, only female samples were used in this study. For presentation purposes, wild-type mCD8::GFP- and rCD2::RFP-positive multi-cellular NB clones are shown in green in all figures. Primary antibodies used in this study include rat monoclonal antibody to mCD8 (1∶100, Invitrogen), rabbit antibody to RFP (1∶500, Clontech), and nc82 (1∶100, DSHB). Secondary antibodies with different fluorophores, Cy3 (Jackson lab), Cy5 (Jackson lab), and Alexa 488 (Invitrogen), were used 1∶200, 1∶200, and 1∶750 dilution in this study. Immunofluorescent signals were collected by Zeiss LSM 710 confocal microscopy and then processed using Adobe Photoshop.

## Supporting Information

Figure S1 **About 32 adPNs are made after birth of GH146-positive adPNs as revealed by dual-expression-control MARCM.** *LexA::GAD-GH146* and *acj6-GAL4* were utilized to label GH146-positive adPNs in magenta and all the adPNs in green in the same NB clones. About 32 green-only adPNs exist in the clone generated in early larvae (A) or even within the one induced during the birth of the last GH146-positive VA1lm-targeting adPN (B). Different focal sections of the AL are shown underneath. Note six glomeruli (VM5d, VM5v, VC3, VC4, DL2d, and DL2v) are exclusively labeled in green and selectively innervated by GH146-negative adPNs. Glomerular identity in this and all other supporting figures was determined based on nc82 immunostaining (blue). The scale bar in this and all other supporting figures equals 10 µm.
(3.60 MB TIF)

Figure S2 **The glomerular pattern of a full-sized adPN clone visualized with** ***GAL4-GH146*** **.** Four focal planes shown in (A) to (D) reveal the glomerular composition of a full-sized adPN clone labeled by *GAL4-GH146* (green). Top panels: GH146-positive adPNs (green); middle panels: nc82 counterstaining (blue); bottom panels: merged images.
(3.00 MB TIF)

Figure S3 **Labeling of a full-sized adPN NB clone and a VP3-targeting single-cell clone by** ***acj6-GAL4*** **.** (A–D) Glomerular targets of the entire adPN lineage are shown in four focal planes. Top panel: labeling of all adPNs by *acj6-GAL4* (green); middle panels: nc82 counterstaining (blue); bottom panels: merged images. (E) An embryonic-born VP3-targeting adPN shown in the regions of the AL (top and middle panels) and the LH (bottom panel). Top and bottom panels: composite confocal images; middle panel: a single focal section.
(4.72 MB TIF)

Figure S4 **Axon projections of differentially marked single-cell clones of adPNs.** Mosaic brains carrying differentially marked single-cell clones of adPNs. Three examples are shown for each specific pair. Note co-migration of neurites in sibling neurons targeting the same glomerulus (A–F). In contrast, distinct paths were taken by sibling neurons that target different AL glomeruli (G, H). It is true even among PNs that have all established a fork-like trajectory (compare [C] and [E] with [H]). In addition, among PNs targeting the same glomerulus (A–F), the detailed trajectories may deviate more between different brains than within a given brain. This might reflect developmental and/or functional plasticity of the brain.
(2.35 MB TIF)

Figure S5 **Stereotyped axon projections of embryonic-born adPNs.** Axon trajectories of previously unidentified embryonic-born adPNs are shown in three different brains for each type. Islets reveal the axons of both single adPNs (magenta) and the accompanying NB clones (green). Note acquisition of analogous projections among adPNs targeting the same glomerulus, including the VM3-targeting adPNs (G and H) that were born in separate windows.
(2.63 MB TIF)

Table S1 **Embryonic-born twin-spot MARCM clones using** ***GAL4-GH146*** **.**
(0.24 MB DOC)

Table S2 **Embryonic-born twin-spot MARCM clones using** ***acj6-GAL4*** **.**
(0.25 MB DOC)

Table S3 **Distribution of cell numbers in adPN twin-spot MARCM clones using** ***GAL4-GH146*** **.**
(0.17 MB DOC)

Table S4 **Distribution of cell numbers in adPN twin-spot MARCM clones using** ***acj6-GAL4*** **.**
(0.12 MB DOC)

Table S5 **Branch number of different adPN types in the MB calyx.**
(0.15 MB DOC)

## Abbreviations

adPN: anterodorsal projection neuron
AL: antennal lobe
CNS: central nervous system
FLP: flipase
GMC: ganglion mother cell
HS: heat shock
iACT: inner antennocerebral tract
LH: lateral horn
LN: local interneuron
MARCM: mosaic analysis with a repressible cell marker
MB: mushroom body
NB: neuroblast
ORN: olfactory receptor neuron
PN: projection neuron
