# Supplementary figures and images for: A Complete Developmental Sequence of a Drosophila Neuronal Lineage as Revealed by Twin-Spot MARCM
Source: PLoS Biol. 2010 Aug 24;8(8):e1000461. doi: 10.1371/journal.pbio.1000461 (PMC2927434; doi:10.1371/journal.pbio.1000461)

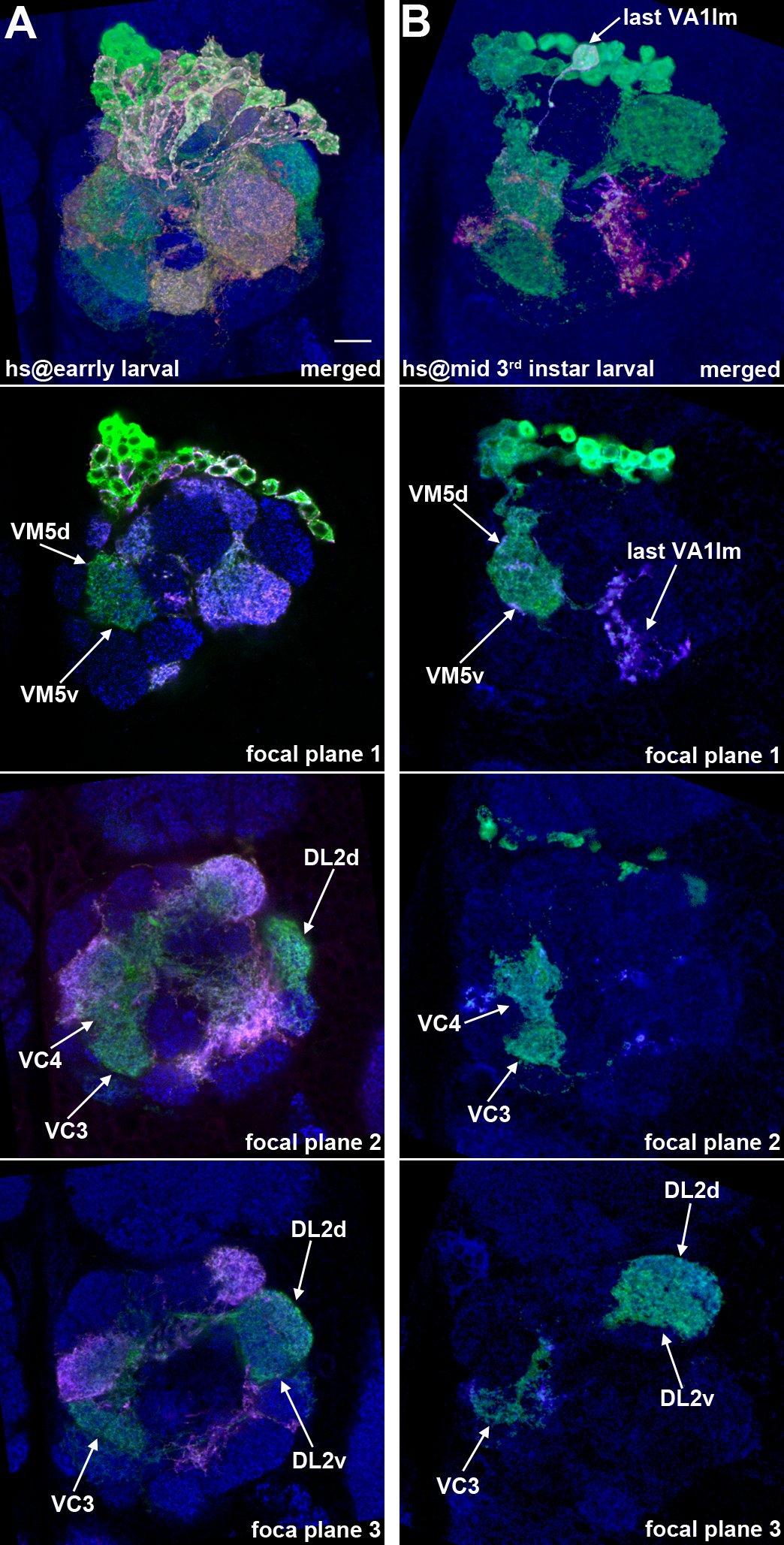

Supplement: Figure S1 — About 32 adPNs are made after birth of GH146-positive adPNs as revealed by dual-expression-control MARCM. LexA::GAD-GH146 and acj6-GAL4 were utilized to label GH146-positive adPNs in magenta and all the adPNs in green in the same NB clones. About 32 green-only adPNs exist in the clone generated in early larvae (A) or even within the one induced during the birth of the last GH146-positive VA1lm-targeting adPN (B). Different focal sections of the AL are shown underneath. Note six glomeruli (VM5d, VM5v, VC3, VC4, DL2d, and DL2v) are exclusively labeled in green and selectively innervated by GH146-negative adPNs. Glomerular identity in this and all other supporting figures was determined based on nc82 immunostaining (blue). The scale bar in this and all other supporting figures equals 10 µm. (3.60 MB TIF) [file pbio.1000461.s001.tif]

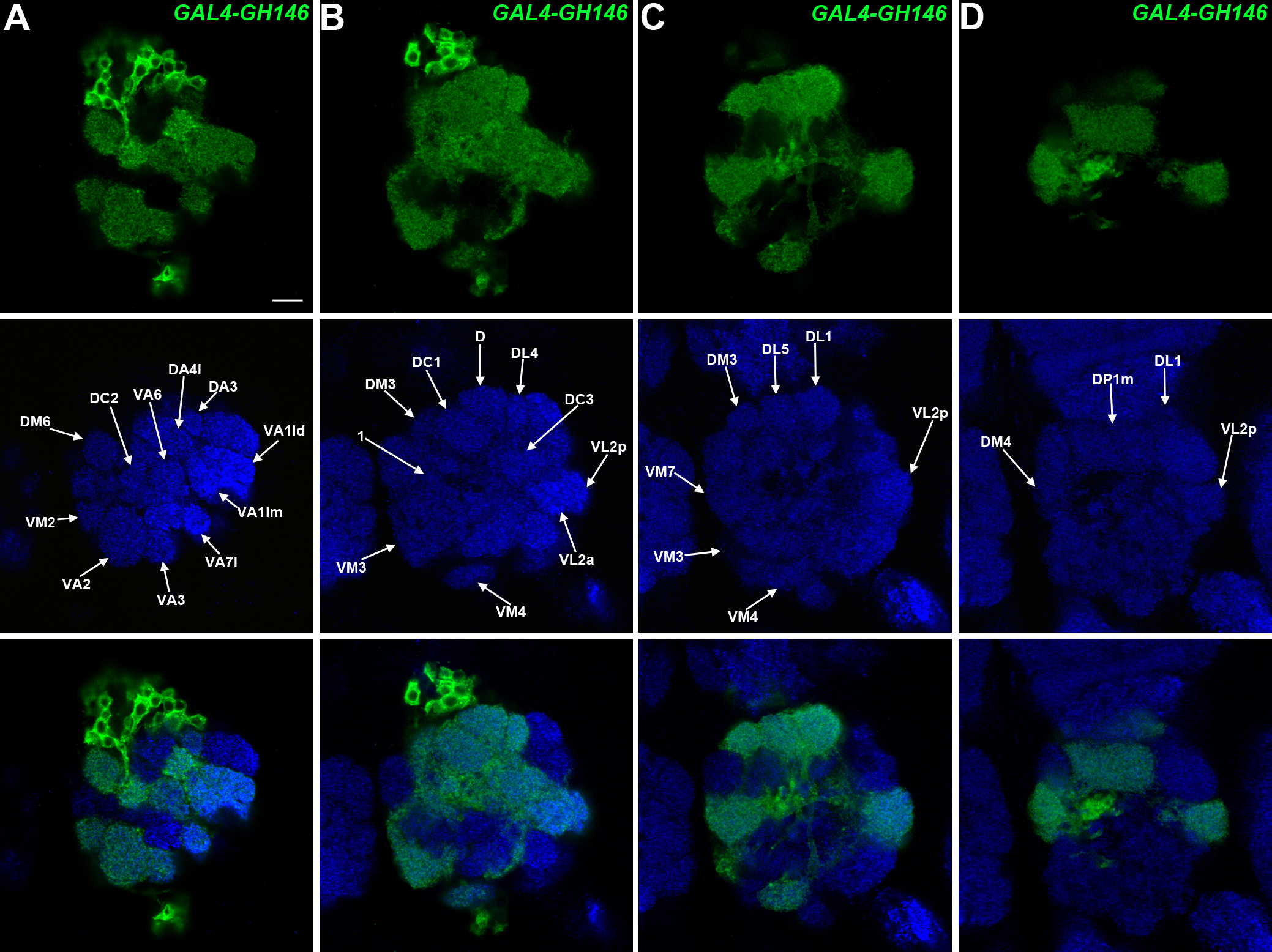

Supplement: Figure S2 — The glomerular pattern of a full-sized adPN clone visualized with GAL4-GH146 . Four focal planes shown in (A) to (D) reveal the glomerular composition of a full-sized adPN clone labeled by GAL4-GH146 (green). Top panels: GH146-positive adPNs (green); middle panels: nc82 counterstaining (blue); bottom panels: merged images. (3.00 MB TIF) [file pbio.1000461.s002.tif]

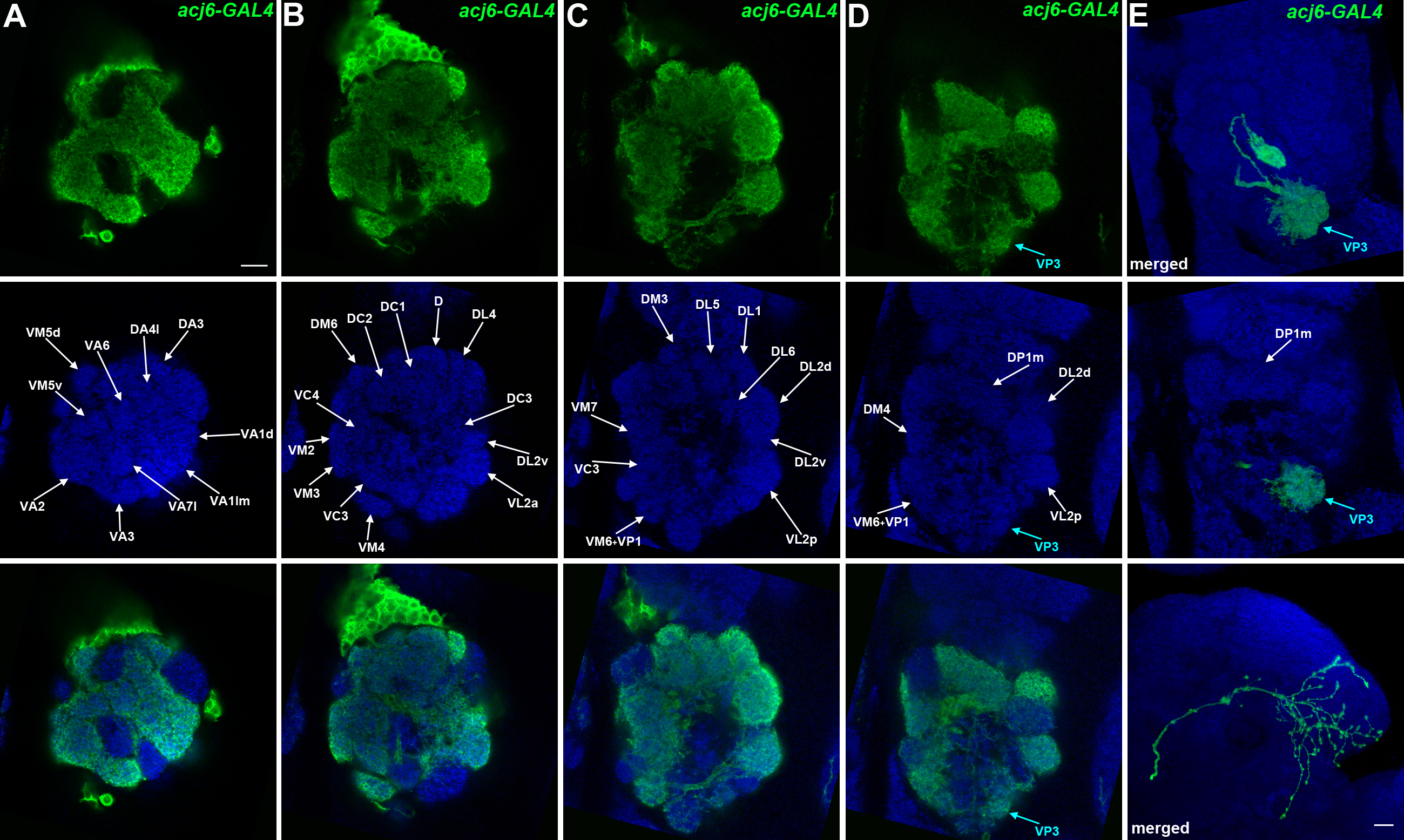

Supplement: Figure S3 — Labeling of a full-sized adPN NB clone and a VP3-targeting single-cell clone by acj6-GAL4 . (A–D) Glomerular targets of the entire adPN lineage are shown in four focal planes. Top panel: labeling of all adPNs by acj6-GAL4 (green); middle panels: nc82 counterstaining (blue); bottom panels: merged images. (E) An embryonic-born VP3-targeting adPN shown in the regions of the AL (top and middle panels) and the LH (bottom panel). Top and bottom panels: composite confocal images; middle panel: a single focal section. (4.72 MB TIF) [file pbio.1000461.s003.tif]

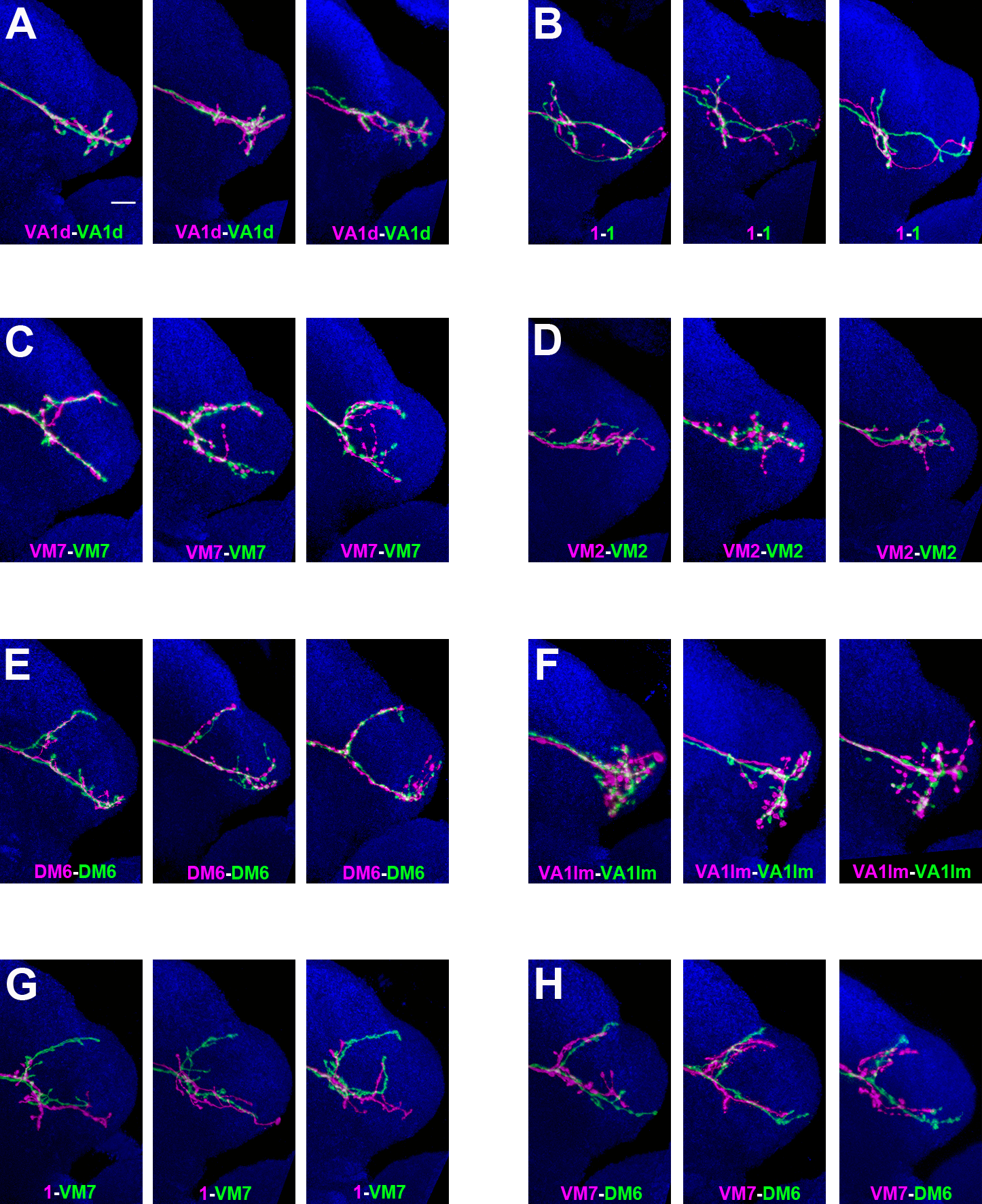

Supplement: Figure S4 — Axon projections of differentially marked single-cell clones of adPNs. Mosaic brains carrying differentially marked single-cell clones of adPNs. Three examples are shown for each specific pair. Note co-migration of neurites in sibling neurons targeting the same glomerulus (A–F). In contrast, distinct paths were taken by sibling neurons that target different AL glomeruli (G, H). It is true even among PNs that have all established a fork-like trajectory (compare [C] and [E] with [H]). In addition, among PNs targeting the same glomerulus (A–F), the detailed trajectories may deviate more between different brains than within a given brain. This might reflect developmental and/or functional plasticity of the brain. (2.35 MB TIF) [file pbio.1000461.s004.tif]

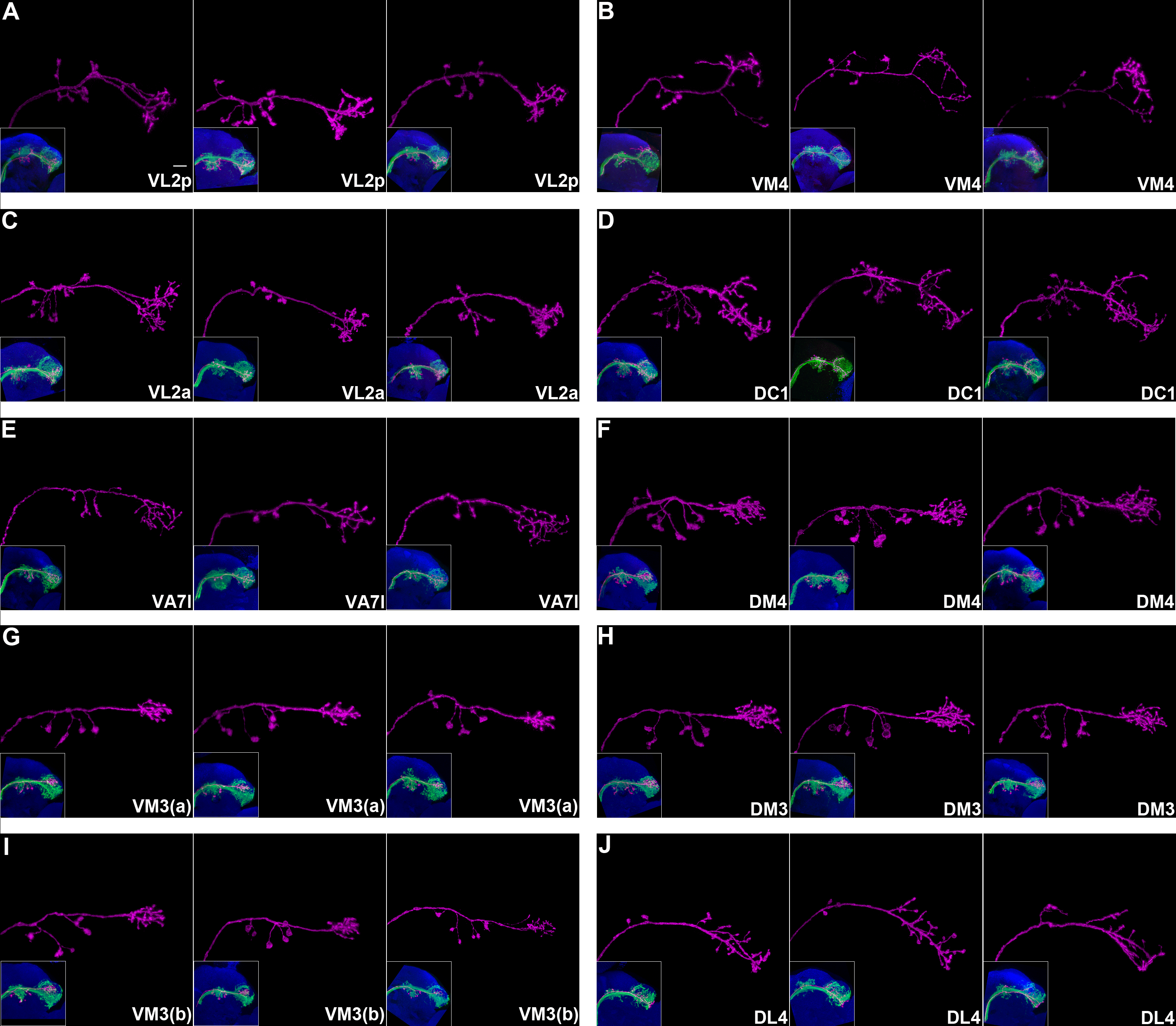

Supplement: Figure S5 — Stereotyped axon projections of embryonic-born adPNs. Axon trajectories of previously unidentified embryonic-born adPNs are shown in three different brains for each type. Islets reveal the axons of both single adPNs (magenta) and the accompanying NB clones (green). Note acquisition of analogous projections among adPNs targeting the same glomerulus, including the VM3-targeting adPNs (G and H) that were born in separate windows. (2.63 MB TIF) [file pbio.1000461.s005.tif]

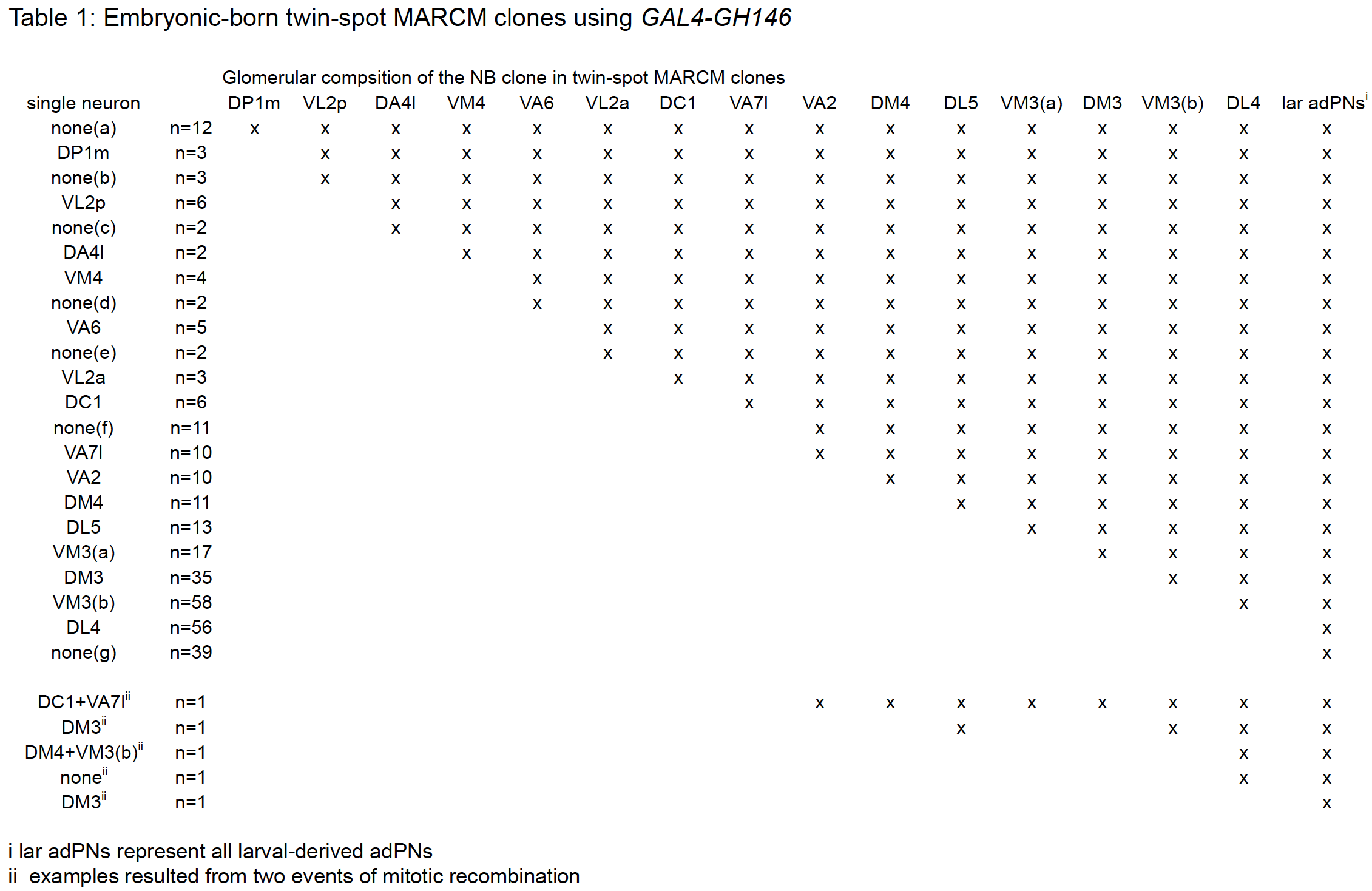

Supplement: Table S1 — Embryonic-born twin-spot MARCM clones using GAL4-GH146 . (0.24 MB DOC) [file pbio.1000461.s006.tif]

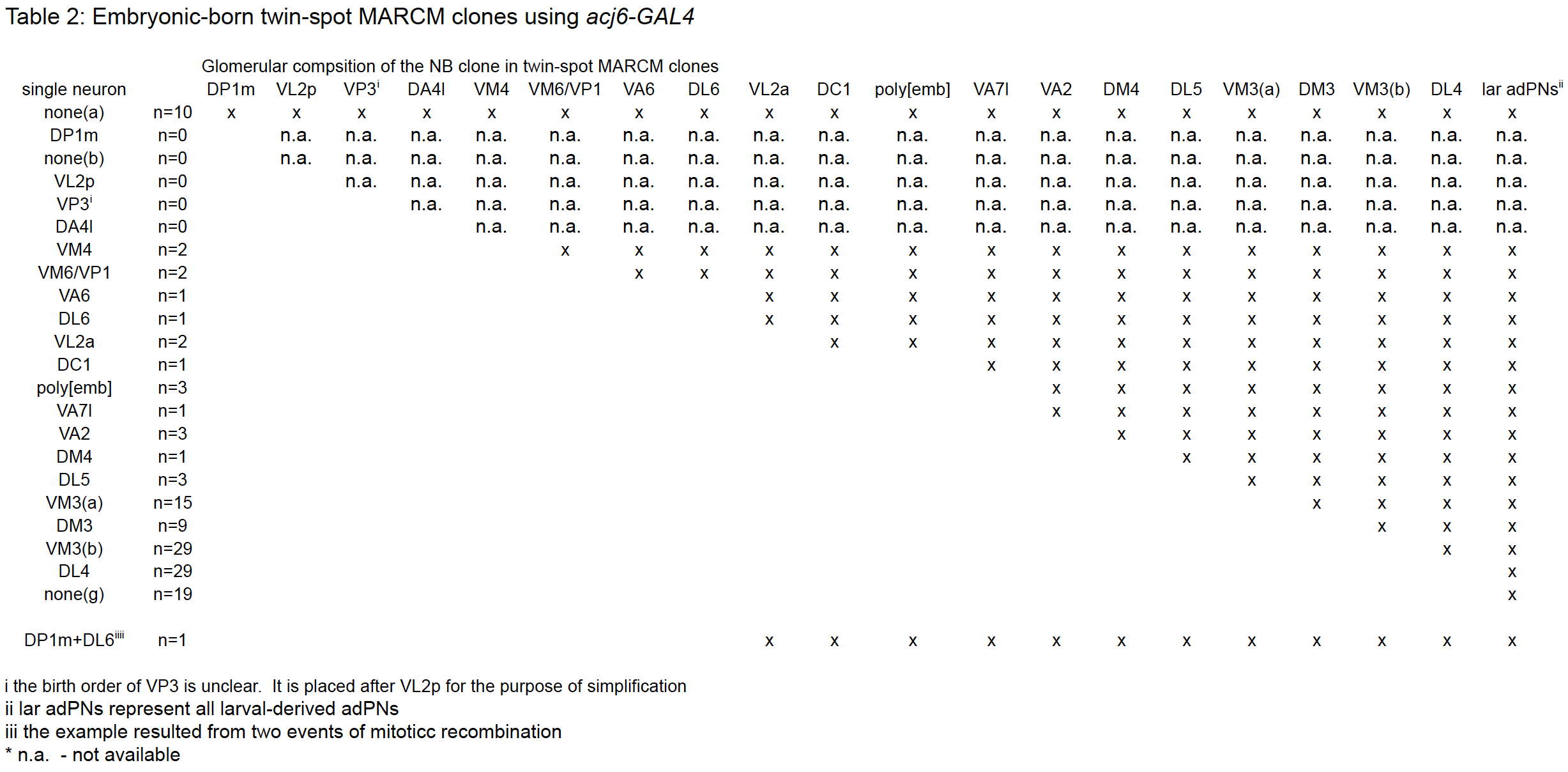

Supplement: Table S2 — Embryonic-born twin-spot MARCM clones using acj6-GAL4 . (0.25 MB DOC) [file pbio.1000461.s007.tif]

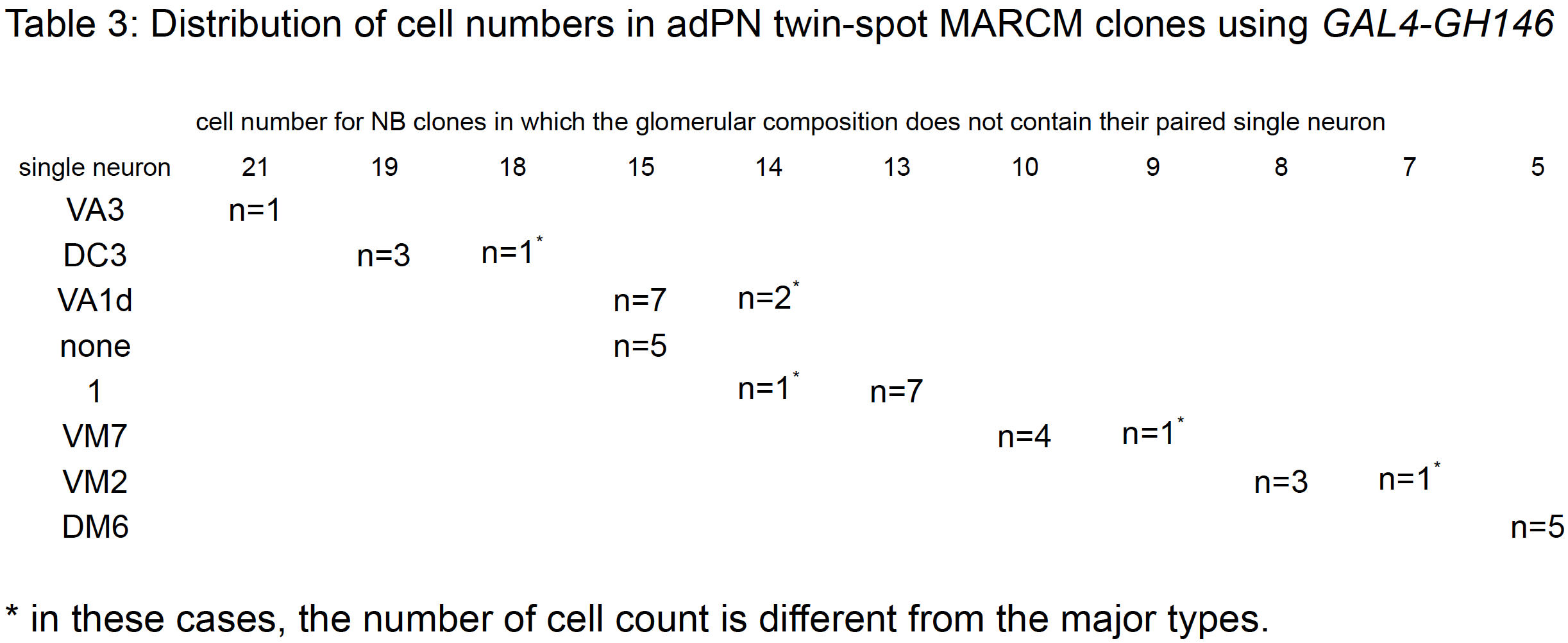

Supplement: Table S3 — Distribution of cell numbers in adPN twin-spot MARCM clones using GAL4-GH146 . (0.17 MB DOC) [file pbio.1000461.s008.tif]

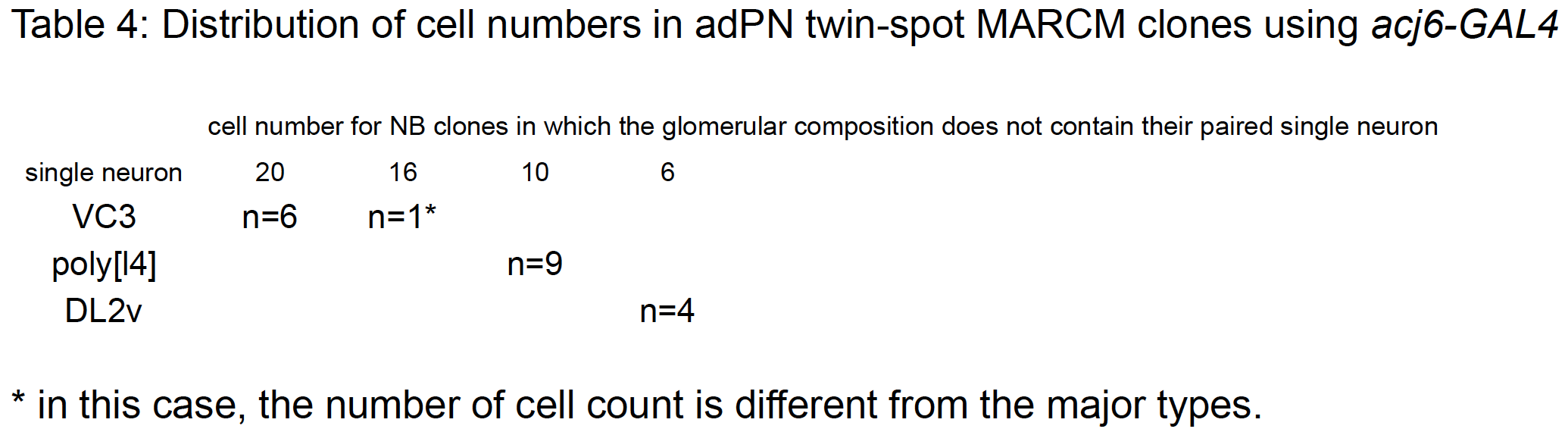

Supplement: Table S4 — Distribution of cell numbers in adPN twin-spot MARCM clones using acj6-GAL4 . (0.12 MB DOC) [file pbio.1000461.s009.tif]

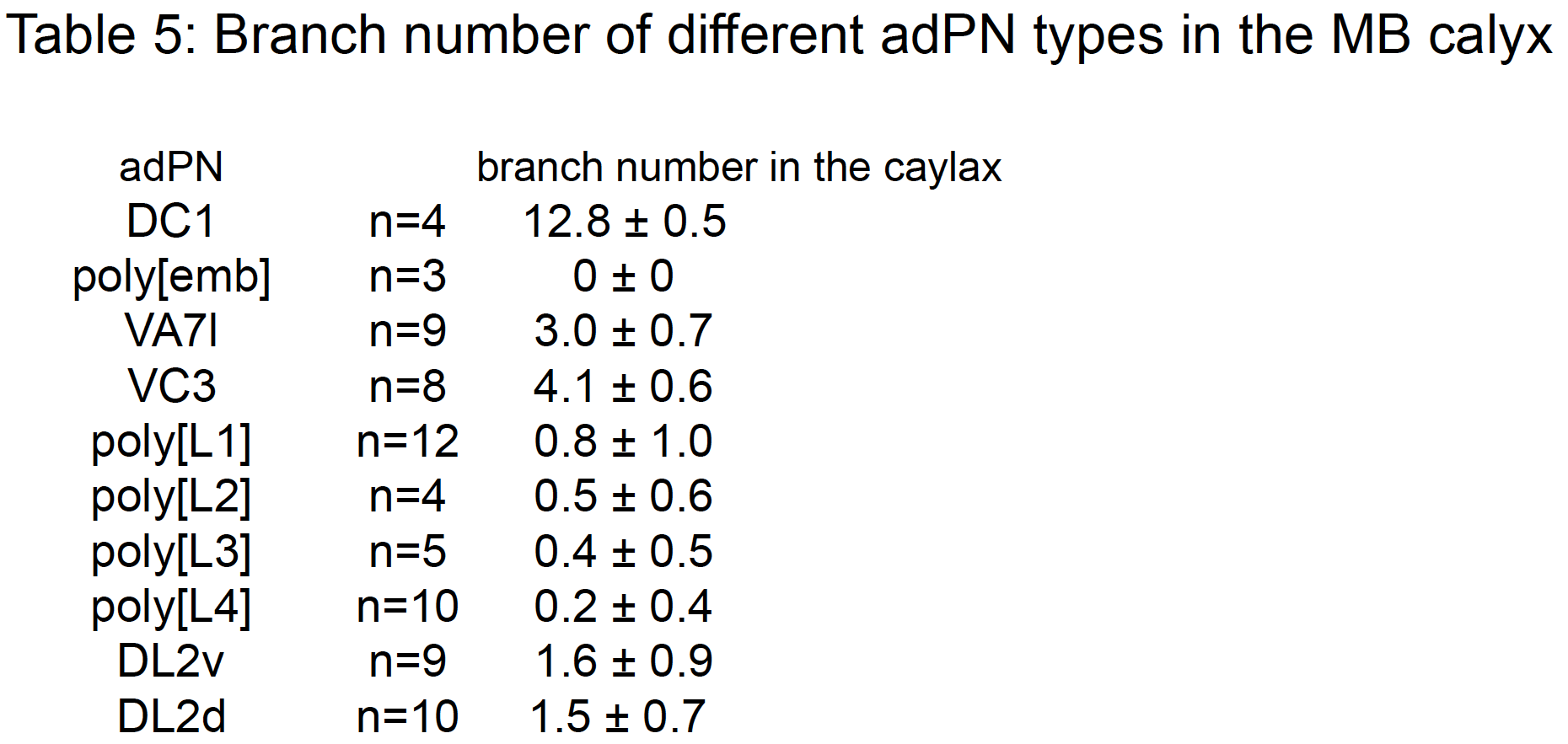

Supplement: Table S5 — Branch number of different adPN types in the MB calyx. (0.15 MB DOC) [file pbio.1000461.s010.tif]
